# Understanding Nosocomial Amplification by Identifying Important Parameters in a Community-Hospital Model

**DOI:** 10.1017/ash.2024.236

**Published:** 2024-09-16

**Authors:** Katelin Jackson, Eric Lofgren

**Affiliations:** Washington State University

## Abstract

**Background:** The phenomena of emerging infectious diseases accelerating once they reach healthcare facilities have been well documented. Outbreaks of MERS-CoV, SARS-CoV, and COVID-19 have led to in-hospital transmission where the initial patient infects healthcare workers, patients, visitors, etc., with infection control policies unable to curtail the spread early on. We refer to this phenomenon as nosocomial amplification. Nosocomial amplification causes an undue burden on a hospital that’s already strained from the pandemic. We aimed to understand which hospital-level parameters impact the community most and vice versa. **Methods:** We adapted an SEIR compartmental model to have two interconnected units, a community unit and a hospital special care unit, to determine the number of COVID-19 acquisitions in each of them over a hypothetical year. The model was stochastically simulated using Gillespie’s Direct Method for 1000 iterations. A parameter sensitivity analysis assessed the effects each parameter had on the model. The original values of all parameters were allowed to vary +/- 50%. The number of simulation acquisitions was normalized as a percent change from the original model’s mean acquisition. **Results:** Our analysis found that parameters impacting the community had a disproportionate impact on COVID-19 acquisitions in the hospital as compared to the special care unit, as did the parameters governing the level of asymptomatic transmission. Transmission between healthcare workers facilitated within-hospital transmission even when strict patient-based cohorting and testing were in place. Extensive community-level transmission was also found to readily overwhelm hospital-level infection control at realistic levels of effectiveness and compliance. **Conclusion:** These findings illustrate that hospitals and the community are tightly linked systems. Hospitals may reintroduce infection into the community that might have contained or mitigated ongoing outbreaks or introduce the disease into a disease-free population; community transmission puts tremendous pressure on infection control. In the future, we can model policies to curb an existing COVID-19 outbreak or subsequent outbreaks to avoid or minimize nosocomial amplification, thus improving the disproportionate burdens on the healthcare system.

